# SPP1 promotes Schwann cell proliferation and survival through PKCα by binding with CD44 and αvβ3 after peripheral nerve injury

**DOI:** 10.1186/s13578-020-00458-4

**Published:** 2020-08-20

**Authors:** Jiang-Bo Wang, Zhan Zhang, Jian-Nan Li, Tuo Yang, Shuang Du, Rang-Juan Cao, Shu-Sen Cui

**Affiliations:** Department of Hand Surgery, China-Japan Union Hospital of Jilin University, 126 Xiantai Street, Changchun, 130033 China

**Keywords:** Peripheral nerve injury, SPP1, PKCα, CD44, αvβ3, Schwann cells, Cell proliferation, Cell apoptosis

## Abstract

**Background:**

Schwann cells (SCs) play a crucial role in Wallerian degeneration after peripheral nerve injury. The expression of genes in SCs undergo a series of changes, which greatly affect the proliferation and apoptosis of SCs as well as the fate of peripheral nerve regeneration. However, how do these genes regulate the proliferation and apoptosis of SCs remains unclear.

**Results:**

SPP1 and PKCα were found upregulated after human median peripheral nerve injury, which promoted SCs proliferation and survival. The promoted proliferation and inhibited apoptosis by SPP1 were blocked after the treatment of PKCα antagonist Gö6976. Whereas, the inhibited proliferation and enhanced apoptosis induced by silence of SPP1 could be rescued by the activation of PKCα, which suggested that SPP1 functioned through PKCα. Moreover, both CD44 and αvβ3 were found expressed in SCs and increased after peripheral nerve injury. Silence of CD44 or β3 alleviated the increased proliferation and inhibited apoptosis induced by recombinant osteopontin, suggesting the function of SPP1 on SCs were dependent on CD44 and β3.

**Conclusion:**

These results suggested that SPP1 promoted proliferation and inhibited apoptosis of SCs through PKCα signaling pathway by binding with CD44 and αvβ3. This study provides a potential therapeutic target for improving peripheral nerve recovery.

## Background

Peripheral nerve injury is a common disability, seriously affecting the quality of daily life [1]. The injured peripheral nervous system often exhibits relatively high regenerative ability compared with the central nervous system, which may partially due to the contribution of Wallerian degeneration [2]. Wallerian degeneration occurs rapidly after peripheral nerve injury [1, 3], characterized by axonal breakdown, myelin degeneration, myelin clearance, and axon regeneration [4]. Schwann cells (SCs), activated at the injured area of the nerve, play an essential role in the Wallerian degeneration process [5, 6]. After the nerve injury, SCs proliferate following dedifferentiation, clearing the myelin debris, forming bands of Büngner to provide a path for re-growing axons, and up-regulating neurotrophic factors [7, 8]. However, during this regeneration process, proliferation and apoptosis will occur in SCs at different times [9, 10]. SCs undergo a series of changes in gene expression, which greatly affect the function of SCs as well as the fate of peripheral nerve regeneration. These genes not only control the proliferation and survival of SCs but also support the survival of injured neurons, which makes them potential targets for therapeutic interventions [11–13]. However, how do these genes control the proliferation and survival of SCs remains unclear, making it difficult to manipulate SCs for therapeutic purposes.

Secreted phosphoprotein 1 (SPP1), also known as osteopontin (OPN), is a secreted glycoprotein with multifunction that influences the adhesion, proliferation, differentiation, migration, and survival of numerous cell types [14–19]. In the peripheral nerve system, SPP1 was identified as a novel SC-expressed gene product that was regulated by axon derived signals [20]. Wright et al. found that SPP1 was upregulated after sciatic nerve transaction and responsible for motor regeneration in rat [21]. However, Küry et al.identified that the expression of SPP1 in rats after sciatic nerve injury was downregulated [22]. Our previous study suggested that the expression of SPP1 was up-regulated after sciatic nerve injury in rats, and elevated SPP1 promoted the proliferation and inhibited the apoptosis of SCs [23]. However, the underlying mechanism is largely unclear. Protein kinase C alpha (PKCα) is downstream of SPP1, and the expression of PKCα is consequently altered when SPP1 is silenced or overexpressed [23]. Furthermore, our early study found that PKCα promoted the proliferation of SCs by activating ERK signaling pathways [24], which led to the hypothesis that SPP1 promoted cell proliferation and inhibited apoptosis of SCs through PKCα signaling pathways.

SPP1 could induce autocrine and paracrine signaling by binding to the cell surface receptors integrin αvβ3 and/or CD44 and transduce cell–matrix signaling into cell to regulates proliferation, survival, migration, and angiogenesis [25–27]. However, CD44 and αvβ3 have their own characteristics when mediating the SPP1 function. Luo et al. reported that SPP1 promoted preneoplastic keratinocyte cellular proliferation and cell survival through CD44 and the activation of the MAPK pathway [28]. Kim et al.found that SPP1 induced osteoclast migration dependent on PI3K signaling via integrin αvβ3 [29]. A recent study showed that microglia promoted the proliferation of neural precursor cells through SPP1-αvβ3 signaling pathway [30]. Although previous studies suggested that SCs expressed both CD44 and αvβ3 on their surface, and responsible for cellular adhesion to extracellular matrix [31, 32], the role of SPP1 in binding to CD44 and/or αvβ3 in Schwann cells has not been investigated. One study has noticed that CD44 played a crucial role in the proliferation and survival of SCs in peripheral nerves during embryonic, and reduced expression of CD44 in SCs led to apoptosis [32]. Therefore, we hypothesize that SPP1 might mediate the functions of SCs by binding to the cell surface receptors αvβ3 and/or CD44.

To clarify this hypothesis, we firstly confirmed the upregulation of SPP1 and PKCα in SCs after peripheral nerve injury in clinical human samples. By primary culture of SCs, SPP1 was found to contribute to the proliferation and inhibit apoptosis of SCs through PKCα. Further, CD44 and αvβ3 were significantly upregulated in SCs of rat after peripheral nerve injury, which consistent with the change of SPP1. EdU and flow cytometry showed that knockdown of CD44 or β3 decreased the proliferation of SCs and increased their apoptosis. Western blot showed that SPP1 regulated the expression of PKCα as well as the downstream cytokines including p-ERK/ERK, Bcl-2/Bax, cleaved Caspase-3/Caspase-3 through αvβ3 and CD44. Our results indicate that SPP1 promotes proliferation and inhibits apoptosis of SCs through PKCα signal transduction by binding to the cell surface receptors αvβ3 and CD44.

## Results

### SPP1 and PKCα were increased in SCs after peripheral nerve injury in human specimens

**Fig. 1:**
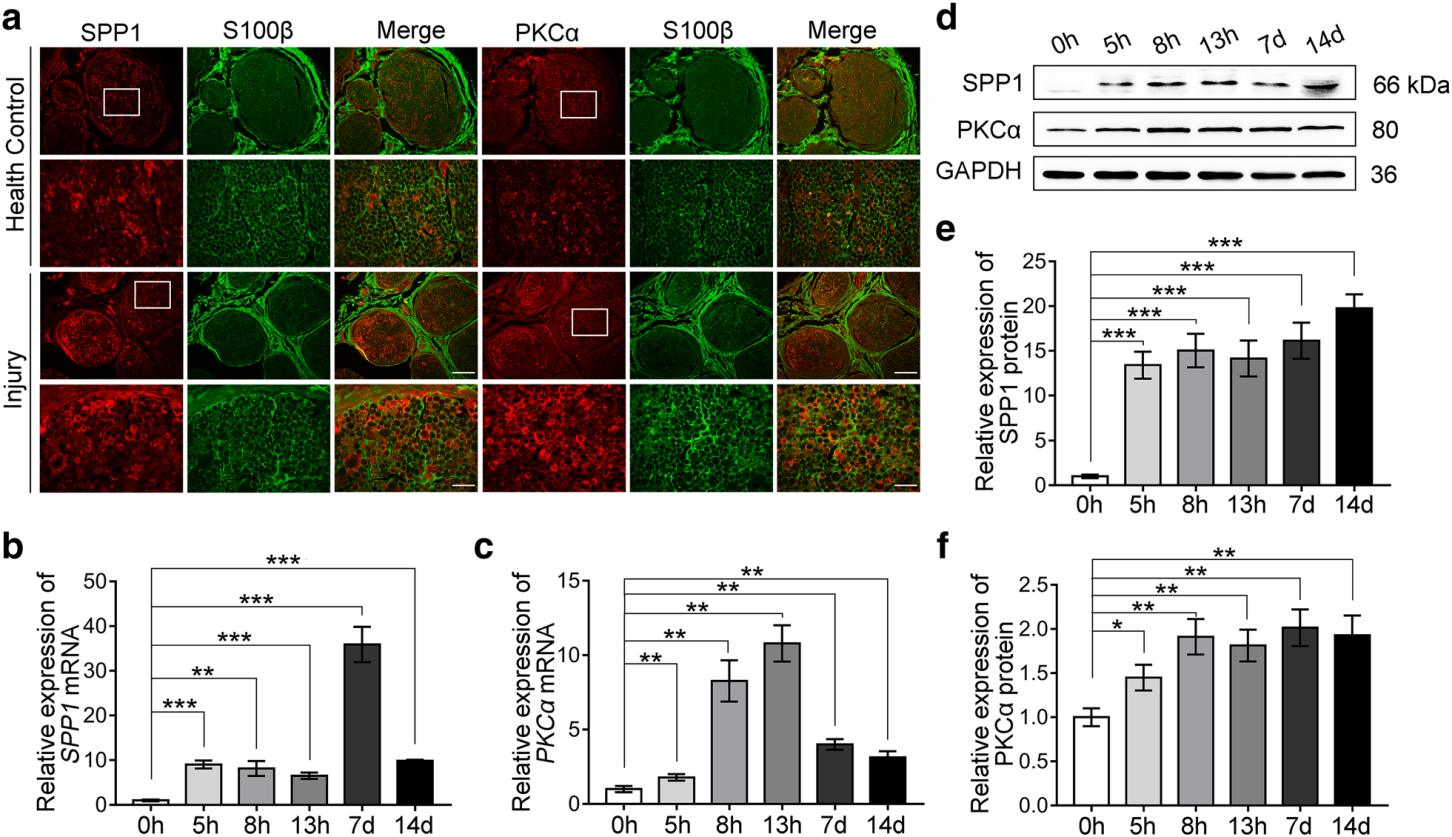
Expression of SPP1 and PKCα was increased in SCs after median nerve injury in the human specimens. **a** Immunofluorescence staining of SPP1 (red), PKCα (red) and S100β (green) in health median nerve and distal median nerve stumps at 5 days after injury. Scale bar, 200 and 50 μm. **b**, **c** Relative mRNA expression of *SPP1* (**b**) and *PKCα* (**c**) in injured median nerve at 0, 5, 8, 13 h, 7, and 14 days of six independent samples by real-time qPCR. **d**–**f** Western blot analysis of SPP1 and PKCα in injured median nerve at 0, 5, 8, 13 h, 7, and 14 days after injury. Repeated for three-time. **d** Representative blot results. **e**, **f** Quantification of the relative expression of proteins in (**d**). Data are presented as mean ± SEM. *P < 0.05, **P < 0.01, ***P < 0.001

In our previous study, we found the expression of SPP1 and PKCα was upregulated after rat sciatic nerve injury [23, 24]. Then, we asked how about this in human peripheral nerve injury. The abandoned human median nerves during surgery were collected (detail in Additional file 1: Table S1) and subjected to the following analysis. Firstly, immunostaining showed that both SPP1 and PKCα were co-localized with S100β positive SCs in the distal nerve stumps 5d after the injury and the expression of SPP1 and PKCα was increased after injury (Fig. 1a). Then, to further quantify and characterize the expression of SPP1 and PKCα, human median nerve-injured 0, 5, 8, 13 h, 7, and 14 days were proceeded to real-time qPCR and Western blot analysis. The relative mRNA expression of *SPP1* increased gradually and reached the peak at 7 days after the injury, then decreased (Fig. 1b). The relative *PKCα* mRNA increased until 13 h after the injury and then dropped at 7 and 14 days (Fig. 1c). For the protein level, both SPP1 and PKCα were gradually increased after injury and maintained at a high expression level as long as 14 days after the injury (Fig. 1d–f). These results suggested that SPP1 and PKCα were upregulated in SCs after the peripheral nerve injury of clinical human specimens.

### PKCα promoted SCs survival in vitro

**Fig. 2:**
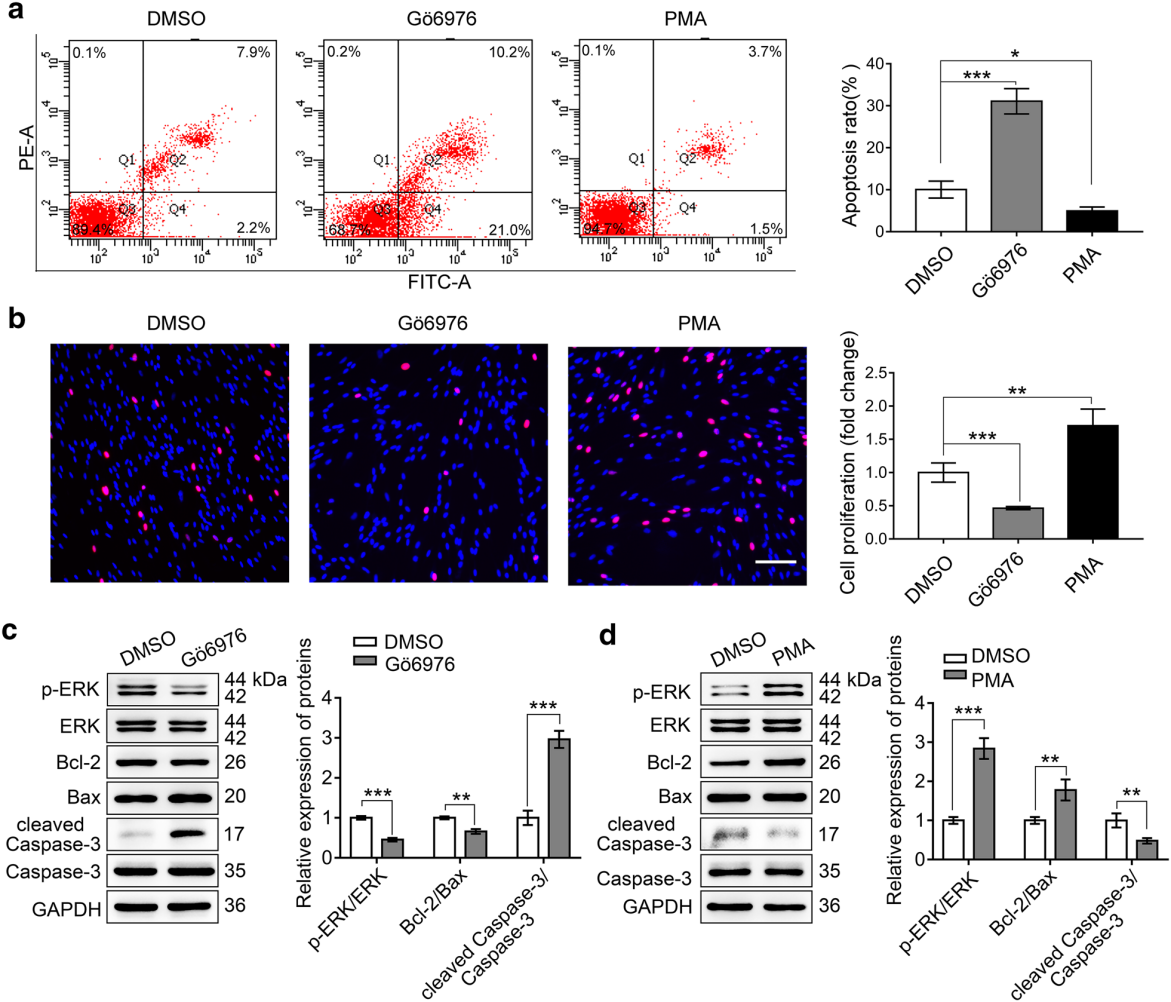
PKCα promoted the proliferation and inhibited apoptosis of SCs. **a** Flow cytometry analyzed the apoptosis of SCs treated by Gö6976 (PKCα inhibitor), PMA (PKCα activator) and DMSO. Representative images (left) and quantification ratio (right) were shown. **b** EdU analysis of the proliferation of SCs treated by Gö6976, PMA, and DMSO. Representative images (left) and quantification data (right) were presented. Scale bar, 100 μm. **c, d** Western blot analysis of p-ERK, ERK, Bcl-2, Bax, cleaved Caspase-3, and Caspase-3 after Gö6976 or PMA treatment in SCs. Representative blot (left) and quantification of relative expression (right) were shown, respectively. Data are shown as mean ± SEM from triplicate experiments. *P < 0.05, **P < 0.01, ***P < 0.001

Increased PKCα was prominent in the SCs and promoted cell proliferation [24], we checked whether PKCα correlated with cell apoptosis. PMA, an activator of PKCα, induces its activation through translocating PKCα from cytosol to membrane [24, 33]. Gö6976 is used as a PKCα inhibitor. With DMSO treatment as control, we examined the effects of PMA (100 nM) and Gö6976 (500 nM) on SCs by flow cytometry and EdU after 12 h treatment. In agreement with our previous reports, we found that the apoptosis of SCs were decreased by PMA and increased by Gö6976 (Fig. 2a). Whereas, SCs proliferation was enhanced by PMA and reduced by Gö6976, compared with the control (Fig. 2b). To further confirm this effect, we analyzed the apoptosis-related proteins after the PMA or Gö6976 treatment by Western blot. The results showed that the inhibition of PKCα activity led to reduced p-ERK/ERK and Bcl-2/Bax, but increased cleaved Caspase-3/Caspase-3 (Fig. 2c). On the contrary, activating PKCα by PMA increased the p-ERK/ERK and Bcl-2/Bax, but decreased cleaved Caspase-3/Caspase-3 (Fig. 2d). These results suggested that PKCα promoted SCs proliferation and survival in vitro*.*

### SPP1 promoted SC proliferation and inhibited apoptosis through PKCα

**Fig. 3:**
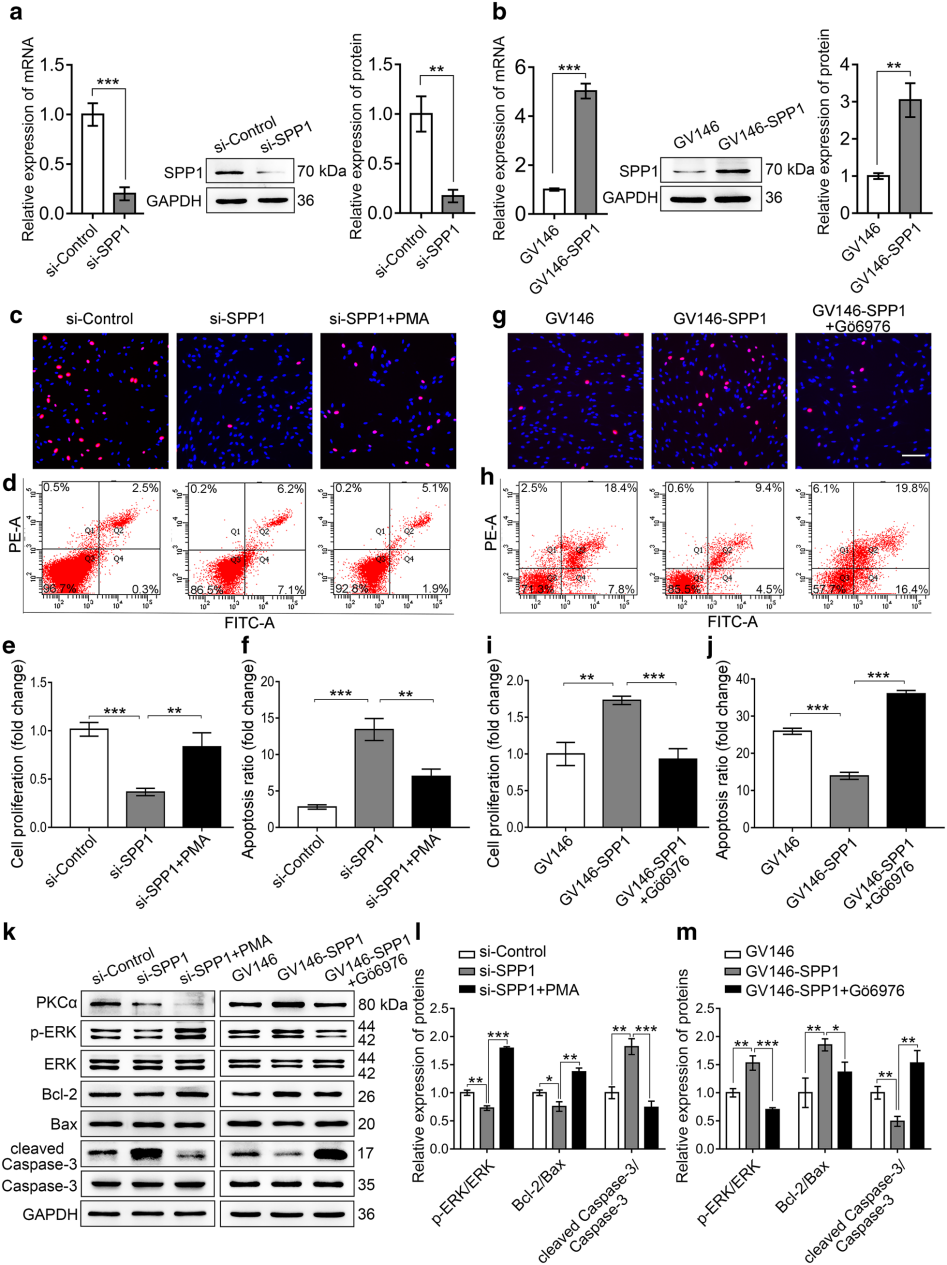
SPP1 promoted SCs proliferation and inhibited the apoptosis through PKCα. **a** Real-time qPCR and Western blot tested the efficiency of SPP1-siRNAs (si-SPP1) in SCs. **b** Real-time qPCR and Western blot examined the overexpression of SPP1 (GV146-SPP1) in SCs. **c**, **e** EdU analysis of the proliferation rate of SCs treated by si-SPP1, si-SPP1 + PMA, and si-Control (**c**), and quantification data was shown (**e**). **d**, **f** The apoptosis of SCs was detected by flow cytometry (**d**), and the apoptosis rate was quantified (**f**). **g**, **i** EdU analyzed the proliferation rate of SCs treated by GV146-SPP1, GV146-SPP1 + Gö6976, and GV146 (**g**). The proliferation of fold change was quantified (**i**). **h**, **j** Flow cytometry tested the apoptosis rate of SCs. **k–m** Western blot analysis of PKCα, p-ERK, ERK, Bcl-2, Bax, cleaved Caspase-3, and Caspase-3 protein expression following si-SPP1, si-SPP1 + PMA transfection and treatment in SCs, or following GV146-SPP1, GV146-SPP1  + Gö6976 transfection and treatment in SCs, respectively. Representative blot (**k**) and quantification of the related expression of proteins (**l**, **m**) were presented. Scale bar, 100 μm. Data were shown as mean ± SEM from three independent experiments. *P < 0.05, **P < 0.01, ***P < 0.001

As both SPP1 and PKCα promoted cell proliferation and inhibited cell apoptosis of SCs, and silence of SPP1 resulted in the reduced PKCα [23], we proposed that SPP1 might function through PKCα. To check this hypothesis, we firstly introduced si-SPP1 to knockdown and GV146-SPP1 to overexpress of SPP1 in primary cultured SCs. Real-time qPCR and Western blot confirmed the silencing efficiency and the overexpression of SPP1 (Fig. 3a, b). Then, whether SPP1 affected SCs proliferation and apoptosis through PKCα was detected. The SCs were transfected with si-SPP1 and 60 h later treated with DMSO or 100 nM PMA for additional 12 h. The proliferation and apoptosis of SCs were then investigated by EdU and flow cytometry. Results showed that the proliferation rate of SCs was significantly reduced and the apoptosis rate was obviously increased by si-SPP1, whereas the PMA-mediated activation of PKCα rescued the inhibited proliferation (Fig. 3c, e) and promoted apoptosis by silence of SPP1 (Fig. 3d, f). Further, the SCs were transfected with GV146-SPP1 and then treated with DMSO or 500 nM Gö6976 for 12 h before analysis. The results showed that enforced expression of SPP1 enhanced the proliferation and decreased the apoptosis rate of SCs. However, treatment with Gö6976 significantly attenuated SPP1-induced cell proliferation (Fig. 3g, i) and inhibited apoptosis (Fig. 3h, j). Besides, the proliferation and apoptosis-related proteins were also analyzed. Western blot showed that silence of SPP1 led to reduced p-ERK/ERK and Bcl-2/Bax, but increased cleaved Caspase-3/Caspase-3. However, this was reversed after the activation of PKCα subsequently (Fig. 3k, l). The overexpression of SPP1 led to increased p-ERK/ERK and Bcl-2/Bax, but decreased cleaved Caspase-3/Caspase-3. Consistently, this effect was disappeared after the inactivation of PKCα (Fig. 3k, m). These data indicated that SPP1 could activated p-ERK/ERK, Bcl-2/Bax and inhibited cleaved Caspase-3/Caspase-3 through PKCα, which responsible for the enhanced proliferation and inhibited apoptosis of SCs.

### CD44 and αvβ3 were upregulated in SCs after sciatic nerve injury in rats

**Fig. 4:**
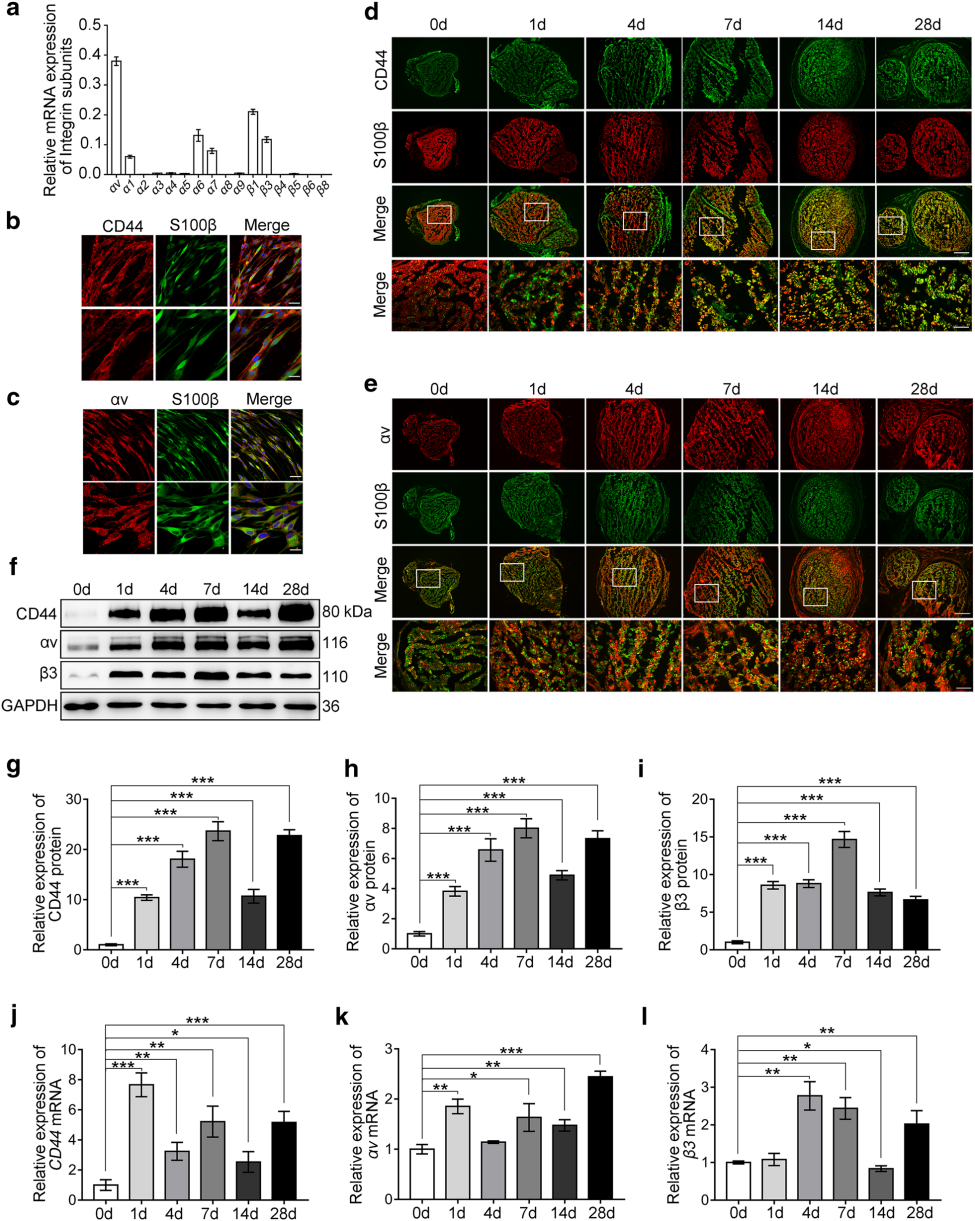
CD44 and αvβ3 were upregulated in SCs after sciatic nerve injury in rats. **a** Real-time qPCR detected the expression of integrin subunits *αv*, *α1*, *α2*, *α3*, *α4*, *α5*, *α6*, *α7*, *α8*, *α9*, and *β1*, *β3*, *β4*, *β5*, *β6*, *β8* in SCs. **b** and **c** Immunofluorescence staining of CD44 (red), αv (red), and S100β (green) in SCs. **b** CD44 mainly located at the cytomembrane. **c** αv mainly concentrated at the focal adhesion. Scale bar, 50 and 25 μm. **d** Immunofluorescence staining of CD44 (green) and S100β (red) in the distal sciatic nerve stumps at 0, 1, 4, 7, 14, and 28 days after injury. Scale bar, 200 and 50 μm. **e** Immunofluorescence staining of αv (red) and S100β (green) in the distal sciatic nerve stumps at 0, 1, 4, 7, 14, and 28 days after injury. Scale bar, 200 and 50 μm. **f–i** Western blot analysis of CD44, αv, and β3 in injured sciatic nerve at 0, 1, 4, 7, 14, and 28 days after injury. **f** Representative blot. **g–i** Quantification of relative expression of proteins in (**f**). **j–l** Real-time qPCR analyzed the relative mRNA expression of *CD44*, *αv*, and *β3* in injured sciatic nerve at 0, 1, 4, 7, 14, and 28 days. Data shown as mean ± SEM from triplicate experiments. *P < 0.05, **P < 0.01, ***P < 0.001

Next, we determined how could SPP1, a secreted protein, function through PKCα. Previous studies have reported that SPP1 interacted with many cell types via various receptors, including CD44 and integrins (αvβ3, αvβ1, αvβ5, αvβ6, α8β1, α5β1, α9β1, and α4β7) [14, 34]. However, receptors have been reported to be expressed on SCs including CD44, αvβ3, αvβ8, α1β1, α2β1, α6β1 and α7β1 [31, 32]. To examine whether CD44 and/or αvβ3 are responsible for SPP1-mediated signaling in Schwann cells. Firstly, we examined the expression of the integrin subunits *αv*, *α1*, *α2*, *α3*, *α4*, *α5*, *α6*, *α7*, *α8*, *α9* and the *β1*, *β3*, *β4*, *β5*, *β6*, *β8* by real-time qPCR. The results showed that *αv* and *β3* were highly expressed in SCs (Fig. 4a). Secondly, immunostaining showed that CD44 was mainly expressed at the cytomembrane of SCs (Fig. 4b) and αv mainly expressed at the focal adhesion in SCs (Fig. 4c). Then, to examine the CD44 and αvβ3 expression in vivo, we set up a sciatic nerve injury Rats model, and distal nerve stumps at 0, 1, 4, 7, 14 and 28 days after injury were collected. The expression of CD44 and αvβ3 was evaluated by immunostaining, Western blot and real-time qPCR analysis. We found that CD44 and αv were co-localized with S100β positive SCs in the distal nerve stumps and the expressions of CD44 and αv were increased after injury (Fig. 4d, e). Western blot and real-time qPCR also showed persistently increasing expression of CD44, αv and β3 in the distal nerve stumps after peripheral nerve injury (Fig. 4f–l), which was consistent with the results of immunostaining. These results suggested that CD44 and αvβ3 receptors were expressed by SCs and significantly increased after peripheral nerve injury.

### CD44 and αvβ3 were involved in promoting SCs proliferation and inhibiting apoptosis

**Fig. 5:**
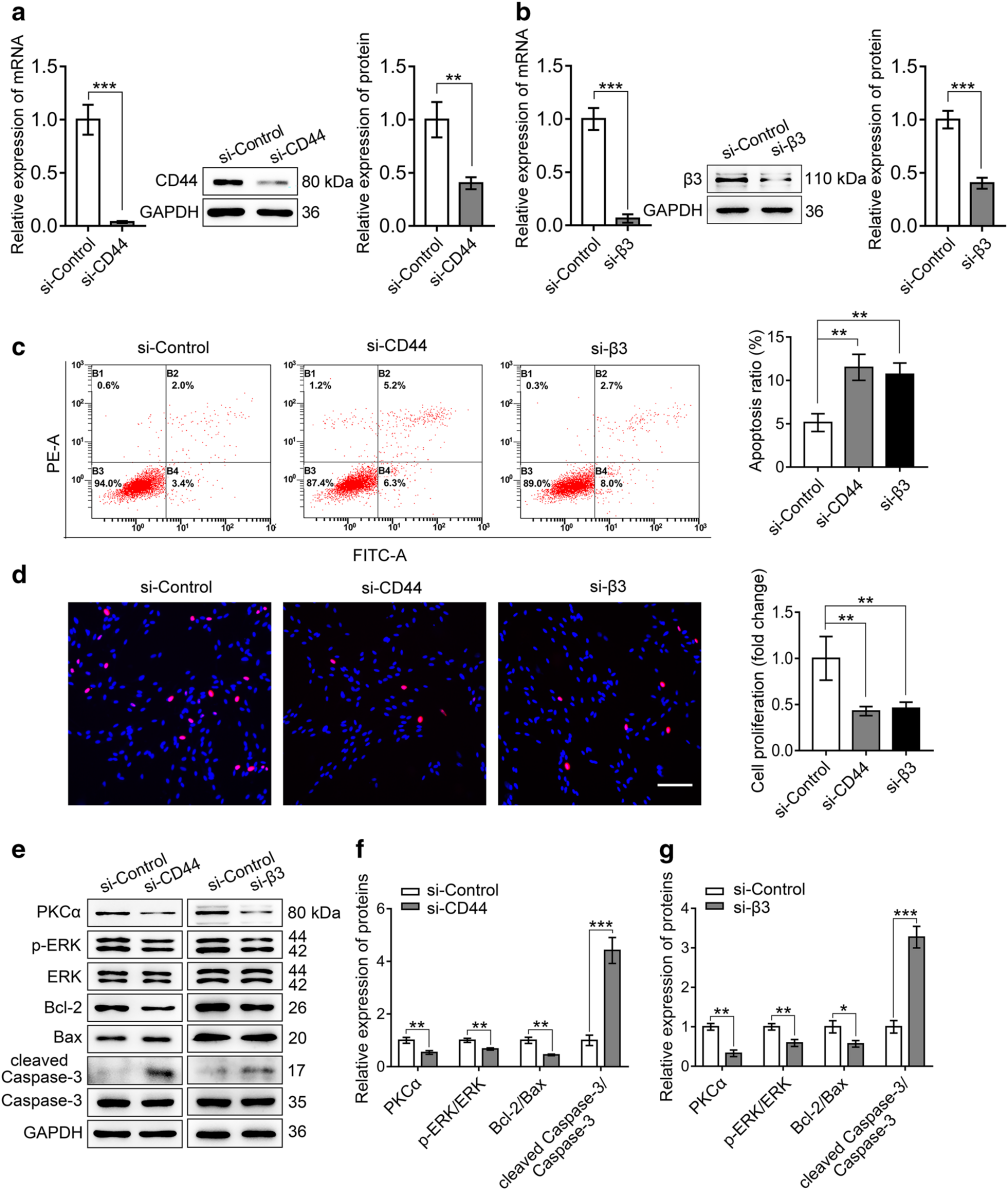
CD44 and αvβ3 were required in promoting SCs proliferation and inhibiting apoptosis. **a**, **b** Real-time qPCR and Western blot detected the efficiency of CD44-siRNA (si-CD44) or β3-siRNA (si-β3) in SCs. **c** Flow cytometry determined the apoptosis rate of SCs. The representative images (left) and quantification data (right) were shown. **d** EdU staining tested the proliferation rate of SCs transfection with si-CD44 or si-β3. Shown are representative images (left) and quantification data (right). Scale bar, 100 μm. **e–g** Western blot analysis of PKCα, p-ERK, ERK, Bcl-2, Bax, cleaved Caspase-3, and Caspase-3 expression (GAPDH serves as loading control) after si-CD44 or si-β3 transfection in SCs. Representative blot (**e**) and quantification of the relative expression of proteins (**f**, **g**) were shown, respectively. Data are obtained from three independent experiments and presented as mean ± SEM. *P < 0.05, **P < 0.01, ***P < 0.001

To investigate whether CD44 and/or αvβ3 were the receptors responsible for SPP1 mediating the proliferation and apoptosis of SCs, siRNAs were designed to silence *CD44* (si-CD44) and *β3* (si-β3), respectively. SCs transfected with si-CD44 or si-β3 were subjected to real-time qPCR and Western blot, and the CD44 or β3 were significantly reduced both at mRNA and protein level (Fig. 5a, b). Then, flow cytometry and EdU analysis were performed with cells transfected by siRNAs. These results showed that the apoptosis was obviously increased in si-CD44 or si-β3 transfected cells (Fig. 5c), whereas the proliferation of the SCs transfected with si-CD44 or si-β3 were reduced (Fig. 5d). Further, to confirm the affected proliferation and apoptosis, the related proteins were analyzed after the transfection of si-CD44 or si-β3. Western blot showed that silence of CD44 or β3 led to reduced PKCα, p-ERK/ERK and Bcl-2/Bax, but increased cleaved Caspase-3/Caspase-3 (Fig. 5e–g). Together, these results suggested that both CD44 and αvβ3 were required for promoting SCs proliferation and inhibiting SCs apoptosis.

### CD44 and αvβ3 were indispensable for SPP1 mediated activation of PKCα and its downstream pathway

**Fig. 6:**
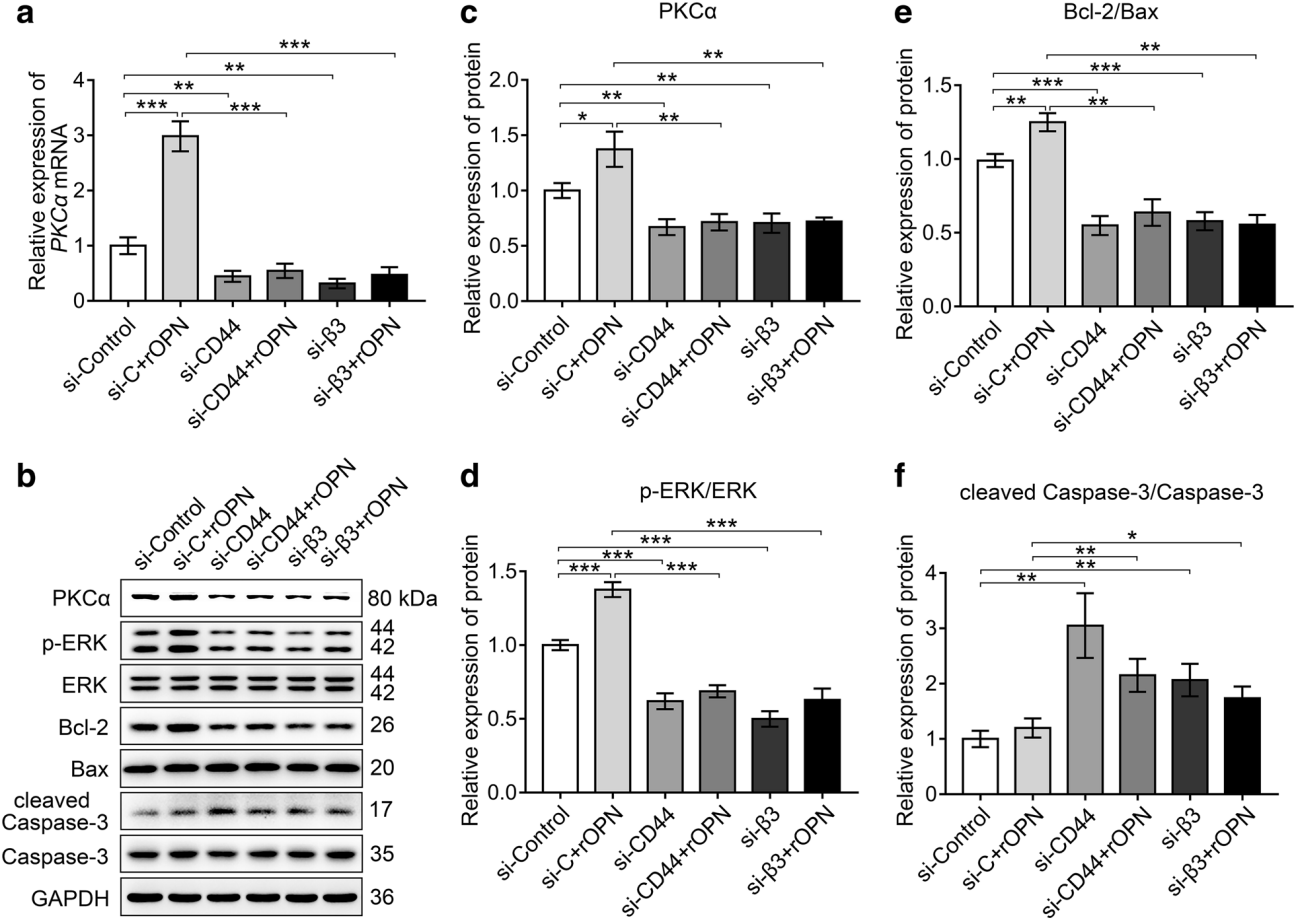
CD44 and αvβ3 were indispensable for SPP1 mediated activation of PKCα and its downstream pathway. **a** Real-time qPCR determined the expression of *PKCα* in SCs transfected with si-CD44, si-β3, or si-Control combined with or without rOPN (50 nM) medium. **b–f** Western blot analysis of PKCα and its downstream cytokines expression levels after siRNA transfection and rOPN treatment in SCs. GAPDH was used as a loading control. **b** Representative blot. **c–f** Quantification of the relative expression of proteins. Data were obtained from three independent experiments and presented as mean ± SEM. *P < 0.05, **P < 0.01, ***P < 0.001

To investigate whether the secreted SPP1 activated PKCα through CD44 and αvβ3, recombinant rat osteopontin (rOPN, 6359OP050, R&D systems) was used to mimic secreted SPP1. Primary SCs were transfected with si-CD44, si-β3, si-Control for 24 h and then incubated with rOPN (50 nM) medium for 48 h. PKCα expression was analyzed by real-time qPCR and Western blot. Results showed an increased PKCα expression by rOPN treatment. However, this increase was blocked by si-CD44 or si-β3, suggesting that binding of SPP1 to CD44 and αvβ3 was required to stimulate PKCα (Fig. 6a–c). It’s worth to notice that the expression of p-ERK/ERK, Bcl-2/Bax was increased by rOPN treatment, but the expression of cleaved Caspase-3/Caspase-3 was not further declined by rOPN treatment. Whereas, the changed expression was significantly alleviated in CD44 and β3 knockdown cells no matter with or without rOPN (Fig. 6b–f). Together, these results suggested that SPP1 promoted proliferation and inhibited apoptosis of SCs through PKCα signal transduction by binding to the cell surface receptors αvβ3 and CD44.

## Discussion

In this study, we reported that SPP1 and PKCα were upregulated after human median peripheral nerve injury in clinic. The elevated PKCα promoted cell proliferation and inhibited apoptosis, which consistent with the function of SPP1 we reported early. Further study found that, the inhibited proliferation and enhanced apoptosis induced by silence of SPP1 could be rescued by the activation of PKCα. Simultaneously, the promoted proliferation and inhibited apoptosis by SPP1 were blocked after the treatment of PKCα antagonist Gö6976, which suggested that SPP1 functioned through PKCα. As SPP1 is a secreted protein, and CD44 and integrins are known receptors for SPP1, we propose that SPP1 functions on SCs through CD44 and/or integrins. To test this hypothesis, CD44 and integrin subunits were analyzed and results showed that both CD44 and αvβ3 were increased after peripheral nerve injury. Silence of CD44 or β3 reduced cell proliferation and increased apoptosis. Furthermore, the function of SPP1 on SCs was blocked if CD44 or β3 was knocked down, suggesting CD44 and αvβ3 were responsible receptors for SPP1 on SCs. Our results provided insight into the mechanism of upregulated SPP1 promoting proliferation and inhibiting apoptosis in SCs after the peripheral nerve injury and identified a novel target for potential therapeutic intervention.

For the difficulty in obtaining biopsy material in individuals, most previous studies on peripheral nerve injury have focused on the rat sciatic nerve. However, some believe that data concerning clinical features is paucity. Here, at the beginning of this study, we checked the expression of SPP1 in human neural specimens. Results showed that both SPP1 and PKCα were upregulated in the distal nerve stumps after median nerve injury. These provided the scientific basis for our following experiment and also provided a theoretical foundation for the clinical transformation of experimental research. Intriguingly, we found that the expression pattern of SPP1 and PKCα after median nerve injury in clinical cases was a little bit different from the performance of the injured sciatic nerve injury in rats [23, 24], but the overall upward trend was consistent. This suggests that there are differences between human and rat, which should be considered in future studies.

In previous study, SPP1, as a promoter of axon regeneration, was expressed by subsets of neurons, such as DRG [35] and axon [36] as well as several classes of glial cells including SCs, oligodendrocytes, microglia [18, 19, 21, 37, 38]. We found that in the median nerve of clinical specimens, SPP1 was not only expressed in myelin sheaths but also existed in axons compared with sciatic nerve in rats. The expression level in axons was significantly downregulated after nerve injury (Fig. 1a), which might due to the degeneration of axons after nerve injury. Further researches are needed to determine the reasons for the downregulation of SPP1 and PKCα in axons after peripheral nerve injury.

Early research showed that SPP1/αvβ3-mediated “outside-in” signaling mediated cell migration via PKCα in the process of osteoclast differentiation [29]. And an review summarized that PKCα promoted cell proliferation by activating ERK and inhibited cell apoptosis by activating Bcl-2 in multiple cell types [39]. Here, we demonstrated that SPP1 contributed to the proliferation and anti-apoptosis of SCs through PKCα. In addition, the underlying molecular mechanisms in regulating the proliferative and anti-apoptotic function of SPP1 through PKCα on SCs were also examined. There are studies indicated that the receptor-mediated ERK1/2 signaling was the essential pathway participating in the proliferative regulation of SCs [40, 41]. Increased ERK1/2 activation would transmit injury signals into nucleus for transcriptional regulation of genes related to SCs differentiation and proliferation [42, 43]. Also, enhanced ERK1/2 expression was observed in regenerating tissues after peripheral nerve injury [44, 45]. Besides, ERK1/2 could be activated by phosphorylation in response to SPP1 [46]and PKCα [47]. In our study, the proliferative effect of SPP1 through PKCα on SCs also found been mediated by ERK1/2 pathway.

Generally, the effect of a factor on cells is multiple, and cell proliferation and apoptosis are concomitant. Damage or death of SCs induced by injuries adversely affects nerve regeneration, and therefore, ensuring the survival of SCs is particularly important for peripheral nerve regeneration. We further investigated the possible mechanisms of SPP1 and PKCα in inhibiting the apoptosis of SCs. Apoptosis signaling occurs through multiple pathways to drive cell death, such as the mitochondrial signaling pathway, death receptors ligation pathway or endoplasmic reticulum stress-mediated signaling pathway [48]. Bcl-2 and Bax, members of the Bcl-2 family, are key regulators of apoptosis. Bcl-2 is an anti-apoptotic factor, while Bax is a pro-apoptotic factor [49]. Bcl-2 binds to Bax to inhibit programmed cell death [50]. Bcl-2, Bax and cleaved Caspase-3 were the main factors involved in the mitochondrial controlled cell apoptosis. It is demonstrated that Bcl-2/Bax, cleaved Caspase-3/Caspase-3 mediated hyperglycemia-induced apoptosis of SCs [51]. In present study, we found that the anti-apoptotic effect of SPP1 through PKCα on SCs was mediated by Bcl-2/Bax-cleaved Caspase-3/Caspase-3 pathway.

SPP1, a member of the matricellular protein family, promoting cell survival and proliferation through autocrine and paracrine in other cells [52–54]. In the peripheral nerve, specific loss of SPP1 in denervated SCs impairs regeneration of motor axons in vivo, indicating that SCs could secrete SPP1 [21]. Autocrine feedback loops are important in the development and function of SCs in that they provide the ability for SCs to survive in the absence of axons [55]. This self-sustainability is an important function in both remyelination and nerve regeneration [56]. By binding to membranous receptors, SPP1 stimulates proliferation and differentiation of NG-2 glial cells into oligodendrocytes by activating the ERK and PI3K/Akt signaling pathways [57]. And other study identified that SPP1 stimulated multiple myeloma cell proliferation through binding to CD44 and was involved in migration through binding to CD44 or αvβ3 [58]. And SPP1 induces angiogenesis through activation of PI3K/AKT and ERK1/2 in endothelial cells by binding to αvβ3 [59]. However, although it has been demonstrated that SPP1 could activate the intracellular signaling pathway through membranous receptors in a variety of cells, the possible existence of SPP1 receptor in SCs as well as the potential involvement of pathway in SCs proliferation and apoptosis have not yet been reported. In this regard, the present study has confirmed that both αvβ3 and CD44 existed in SCs as SPP1 receptors, and responsible for SPP1 and its downstream in inducing cell proliferation and anti-apoptosis.

## Conclusion

In summary, our data suggested that SPP1 and PKCα were significantly upregulated after peripheral nerve injury in clinical specimens. SPP1 promoted proliferation and inhibited apoptosis of SCs through PKCα signaling pathway by binding with CD44 and αvβ3. PKCα-ERK1/2 signaling is a major signaling pathway involved in the proliferative regulation of SPP1 on SCs. The anti-apoptotic effect of SPP1 through PKCα on SCs has been demonstrated to be mediated by Bcl-2/Bax pathway. Further research is needed to elucidate the contribution of SPP1 in the following axon regeneration and functional recovery after peripheral nerve injury.

## Materials and methods

### Human samples

Clinical specimens were collected in accordance with the guidelines of the ethics committee of China-Japan Union Hospital of Jilin University and the World Medical Association Declaration of Helsinki. Informed consents were obtained from all the patients recruited randomly in this study. Inclusion criteria: the median nerve was completely severed as a result of acute injury to the upper extremity. Exclusion criteria: avulsion of the nerve, previous peripheral neuropathy, and other systemic diseases that could cause neuropathy, such as diabetes. The distal nerve stumps of median nerve from patients range in age from 18 to 60, were obtained from China-Japan Union Hospital of Jilin University from January 2018 to April 2020, totally 8 cases. The median nerve tissues injured after 0 h to 14 days were discarded during upper limb injury debridement in the first stage and second stage. The control tissues were obtained from the redundant uninjured nerve tissue of emergency amputation patients who underwent stump trimming during surgery. Under the principle of no harm to the patient, the specimen size was determined according to the intraoperative conditions.

### Animal model

The protocols and procedures involving rats were approved by the Institutional Animal Care and Use Committee of Jilin University in accordance with NIH Guidelines for the Care and Use of Laboratory Animals. Rats were provided by the Animal Center of Jilin University. Male Sprague–Dawley rats weighing 180–220 g were selected and randomly divided into six groups (eight rats per group) and underwent sciatic nerve transection [60]. The rats were anesthetized by isoflurane using a small animal anesthesia apparatus (RWD, Jiangsu, China). The left sciatic nerve was cut and a 5-mm segment was excised. Rats were sacrificed immediately after the experiment (0 day), or 1, 4, 7, 14 and 28 days after the surgery.

### Primary culture of SCs

SCs were isolated from the sciatic nerves obtained from dissected sciatic nerves of 2-day-old rats, minced, incubated in 3 mg/mL collagenase for 30 min at 37 °C, followed by trypsinization at 37 °C for 8 min. Primary cultures of SCs were maintained in Dulbecco’s modified Eagle’s medium (DMEM) supplemented with 10% fetal bovine serum (FBS), 100 IU/mL penicillin, 100 g/mL streptomycin at 37 °C in a 5% CO_2_ humidified atmosphere. Primary cultures were then treated with cytosine β-D-arabinofuranoside (C1768, Sigma-Aldrich) at 10 μM to remove fibroblasts. The viable fibroblasts were eliminated by complement cleavage of polyclonal anti-Thy1.1 antiserum (1:1000, M7898, Sigma-Aldrich) and rabbit complement (234,400, EMD Millipore). The final cells consisted of 98% SCs were determined by immunofluorescence for mouse anti-S100β (S2532, Sigma-Aldrich) monoclonal antibody which is a specific SC marker.

### Immunofluorescence

Immunofluorescence was used to visualize the location of SPP1, PKCα, CD44, αv, β3 and S100β in cultured SCs and nerve samples. SCs and nerve samples were fixed in pre-cold 4% paraformaldehyde (PFA). The nerve samples were dehydrated in 30% sucrose solution. The transverse sections (12 μm thickness) were taken at 7 mm distal from the injury site using a cryostat microtome (CM1950, Leica, Germany). Tissues and SCs were subsequently permeabilized in 0.5% Triton X-100 at room temperature, washed three times by 0.01 M PBS, and blocked with 5% BSA in PBS containing 0.2% Tween 20. Then, tissues or SCs were incubated with primary antibodies. Reaction products were visualized by staining with secondary antibodies labeled with Alexa-488 and Alexa-546 (listed in Additional file 1: Table S2). Cell nuclei were counterstained with DAPI (1:5000, C1002, Beyotime) for 10 min. Fluorescence microscope (DM4B, Leica, Germany) and laser scanning confocal microscopy (A1HD25, Nikon, Japan) were used to capture images.

### Small interfering RNA (siRNA) transfection

RNA interference (RNAi) was performed using small interfering RNAs (siRNAs). SCs were transfected with siRNAs including SPP1 siRNA, CD44 siRNA or β3 siRNA (Integrated Biotech Solutions, Shanghai, China) using Lipofectamine 3000 transfection reagent (L3000150, Invitrogen) according to the manufacturer’s instructions. The medium was replaced with DMEM supplemented with 10% FBS 6 h after the transfection. The negative control was designed as a non-specific sequence. The sequences of siRNA are listed, SPP1: 5′-AGCUAGUCCUAGACCCUAA-3′, CD44: 5′-GAGUCAAGAGGAUGUUUC-3′, β3: 5′-GCUUCAAUGACGAAGUGAA-3′, non-specific control siRNA: 5′-UUCUCCGAACGUGUCACGU-3’.

### Overexpression of SPP1 in SCs

The SPP1 overexpression plasmid GV146-SPP1 was constructed and confirmed by GeneChem (Shanghai, China). Shortly, mouse full-length SPP1 was cloned into expressing vector GV146 between XhoI and EcoRI restriction enzyme sites. SCs were transfected with GV146-SPP1 using Lipofectamine 3000 transfection reagent (L3000150, Invitrogen). GV146 plasmid served as control. Real-time qPCR was performed 48 h after the transfection and Western blot was performed 72 h after the transfection. Each experiment was repeated three times.

### PKCα inhibition and activation

The PKCα inhibitor Gö6976 (365,250) and activator Phorbol-12-myristate-13-acetate (PMA, P8139) were obtained from Sigma-Aldrich and dissolved in dimethylsulfoxide (DMSO). The SCs were incubated with medium containing 100 nM PMA or 500 nM Gö6976 for 12 h, and medium with DMSO served as control. SCs were then transferred to microwell plates for further analysis.

### Real-time qPCR analysis

The Eastep™ Super Total RNA Extraction Kit (LS1040, Promega) was used to extract total RNA and TrancScript One-Step cDNA Synthesis SuperMix (AT311, TransGen Biotech) was used to synthesize cDNA. The primers were synthesized by Genewiz Biotech (listed in Additional file 1: Table. S3). cDNA templates and primers were mixed with TB Green™ Premix Ex Taq™ (RR420A, TaKaRa), and the real-time quantitative PCR was performed with Real-Time PCR System (CFX96, Bio-Rad). The relative expression was calculated using the 2^−ΔΔCt^ method. GAPDH was used as an endogenous control.

### Western blot analysis

SCs or 2 mm injured nerve samples were lysed in RIPA buffer containing 1 × protease inhibitor cocktails (C50008, Sangon Biotech). Protein concentrations were measured with a BCA assay (P0010, Beyotime Biotechnology). Protein samples were separated by 10% SDS-PAGE and transferred to PVDF membranes. The membranes were blocked with 5% BSA in TBST for 1.5 h at room temperature and then incubated with the primary antibodies at 4 °C overnight. Lastly, the membranes were incubated with the secondary antibodies (1:1000, Beyotime) for 2 h at room temperature. Protein expression was analyzed with antibodies against SPP1, PKCα, AKT, p-AKT, ERK, p-ERK, Bcl-2, Bax, Caspase-3, cleaved Caspase-3 and GAPDH (listed in Additional file 1: Table. S2). The images were scanned with the GS800 Densitometer Scanner. PD Quest 7.2.0 software was used to analyze the optical density. GAPDH was used as a control to normalize the protein levels. Experiments were repeated three times with fresh samples and representative pictures were shown.

### Cell proliferation assay

Cell proliferation was assessed after cell transfection. EdU solution (1:1000) was added to the cell culture and incubated for 4 h. The SCs were then fixed with 4% PFA for 30 min. After SC labeling, a Cell-Light EdU DNA Cell Proliferation Kit (C10310, Ribobio) was used to analyze cell proliferation according to the manufacturer’s protocol. Cell proliferation was shown as the ratio of EdU-positive cells, which was quantified with images of randomly selected on a DMR fluorescence microscope (Ts2R-FL, Nikon, Japan).

### Flow cytometry analysis

SCs apoptosis was measured using the Annexin V-FITC Apoptosis Detection Kit (BD Biosciences) according to the manufacturer’s instruction. Briefly, SCs were collected after washed with PBS. Cells were stained with 5 μL FITC-labeled annexin V and 195 μL binding buffer for 10 min at room temperature and incubated with 10 μL propidium iodide 10 min on ice in the dark. FACScan flow cytometry was used to measure the apoptotic cells.

### Statistical analysis

Statistical analysis was performed using SPSS 15.0 for windows (SPSS, Inc, Chicago, IL, USA). Unpaired student’s t-test was used for comparisons between two groups. Values of P less than 0.05 were considered statistically significant. All experiments were performed independently at least three times. All data were shown as mean ± SEM.

## Supplementary information

**Additional file 1: Table. S1.** The details of Clinical specimen; **Table. S2.** Antibodies used in the experiment; **Table. S3.** Sequences of the primers for Real-time qPCR.

## Data Availability

All data generated or analyzed during this study are included in this published article (and its additional files).

## Abbreviations

SCs: Schwann cells
SPP1: Secreted phosphoprotein 1
OPN: Osteopontin
PKCα: Protein kinase C alpha
rOPN: Recombinant rat osteopontin
DMEM: Dulbecco’s modified Eagle’s medium
FBS: Fetal bovine serum
PFA: Paraformaldehyde
siRNA: Small interfering RNA
RNAi: RNA interference
siRNAs: Small interfering RNAs
