# Supplementary material for: SPP1 promotes Schwann cell proliferation and survival through PKCα by binding with CD44 and αvβ3 after peripheral nerve injury
Source: Cell Biosci. 2020 Aug 20;10:98. doi: 10.1186/s13578-020-00458-4 (PMC7439540; doi:10.1186/s13578-020-00458-4)
Supplement: Supplementary file 1 — Additional file 1: Table. S1. The details of Clinical specimen; Table. S2. Antibodies used in the experiment; Table. S3. Sequences of the primers for Real-time qPCR. [file 13578_2020_458_MOESM1_ESM.docx]

**Table.S1. The details of Clinical specimen**

| Human specimen | Age（years） | Gender | Injuried upper limbs | Dating of injury | Body mass index(kg/m^2^) | Application |
| --- | --- | --- | --- | --- | --- | --- |
| 1 | 45 | Male | Right | 7d | 25 | WB, Real-time qPCR |
| 2 | 63 | Male | Left | 5h | 26 | WB, Real-time qPCR |
| 3 | 45 | Male | Right | 7h | 24 | WB, Real-time qPCR |
| 4 | 45 | Male | Right | 13h | 27 | WB, Real-time qPCR |
| 5 | 42 | Male | Right | 14d | 23 | WB, Real-time qPCR |
| 6 | 50 | Male | Right | 0h | 19 | WB, Real-time qPCR |
| 7 | 52 | Male | Left | 5d | 22 | IF |
| 8 | 38 | Male | Left | 0d | 23 | IF |

**Table. S2.** **Antibodies used in the experiment**

| Antibody | Applications/Dilution | Catalog | Company |
| --- | --- | --- | --- |
| SPP1 | WB 1:1000  IF 1:1000 | ab8448 | Abcam |
| PKCα | WB 1:1000  IF 1:100 | #2056 | Cell Signaling Technology |
| ERK | WB 1:1000 | #4695 | Cell Signaling Technology |
| p-ERK | WB 1:1000 | #4370 | Cell Signaling Technology |
| Bcl-2 | WB 1:1000 | ab59348 | Abcam |
| Bax | WB 1:1000 | ab32503 | Abcam |
| Caspase-3 | WB 1:100 | AB1899 | Sigma-Aldrich |
| cleaved Caspase-3 | WB 1:1000 | #9664 | Cell Signaling Technology |
| CD44 | WB 1:1000  IF 1:1000 | #5640S | Cell Signaling Technology |
| β3 | IF 1:1000 | ab119992 | Abcam |
| αv | WB 1:5000  IF 1:500 | Ab179475 | Abcam |
| S100β | IF 1:500 | S2532 | Sigma-Aldrich |
| S100β | IF 1:100 | ab52642 | Abcam |
| GAPDH | WB 1:5000 | HC301 | TransGen Biotech |
| anti-Mouse IgG (H+L) labeled with Alexa-488 | IF 1:1000 | A32766 | Invitrogen |
| Anti-rabbit IgG (H+L) labeled with Alexa-488 | IF 1:1000 | A11008 | Invitrogen |
| Anti-Mouse IgG(H+L) labeled with Alexa-546 | IF 1:500 | A10040 | Invitrogen |
| Anti-Rabbit IgG(H+L) labeled with Alexa-546 | IF 1:500 | A11035 | Invitrogen |

**Table.S3.** **Sequences of the primers for Real-time qPCR**

| Gene | Sequences | Organism |
| --- | --- | --- |
| *GAPDH* | Forward: 5’ GTCTCCTCTGACTTCAACAGCG 3’  Reverse: 5’ ACCACCCTGTTGCTGTAGCCAA 3’ | Homo sapiens |
| *SPP1* | Forward: 5’ CAGTGATTTGCTTTTGCCTCC 3’  Reverse: 5’ GGCTAGGAGATTCTGCTTC 3’ | Homo sapiens |
| *PKCα* | Forward: 5’ GCCTATGGCGTCCTGTTGTATG 3’  Reverse: 5’ GAAACAGCCTCCTTGGACAAGG 3’ | Homo sapiens |
| *GAPDH* | Forward: 5’ TGGAGTCTACTGGCGTCTT 3’  Reverse: 5’ TGTCATATTTCTCGTGGTTCA 3’ | Rattus norvegicus |
| *SPP1* | Forward: 5’ AACAGTATCCCGATGCCACA 3’  Reverse: 5’ TTCCCGTTGCTGTCCTGAT 3’ | Rattus norvegicus |
| *PKCα* | Forward: 5’ GAACACATGATGGACGGGGTCACGAC 3’  Reverse: 5’ CGCTTGGCAGGGTGTTTGGTCATA 3’ | Rattus norvegicus |
| *CD44* | Forward: 5’ CGAAGAAGAGGTGGAAGTC 3’  Reverse: 5’ GGAGTCAGTAGCAACAGTC 3’ | Rattus norvegicus |
| *Integrin αv* | Forward: 5’ ATGAACAAGGAGAACCAGAA 3’  Reverse: 5’ GAGAACCGCCAAGATGAT 3’ | Rattus norvegicus |
| *Integrin α1* | Forward: 5’ CTAAGCAGACACAGGTCGGG 3’  Reverse: 5’ AGGGCTGTCATCGTTTGGAG 3’ | Rattus norvegicus |
| *Integrin α2* | Forward: 5’ TGACAACCGAATGGGAGACG 3’  Reverse: 5’ AAACCCAGGCTCATGTTGGT 3’ | Rattus norvegicus |
| *Integrin α3* | Forward: 5’ ACATGGTTCTCCGTGGACAT 3’  Reverse: 5’ CTTAGCTTCATACAGGGCACGA 3’ | Rattus norvegicus |
| *Integrin α4* | Forward: 5’ CTGTTTGGCTACTCGGTGGT 3’  Reverse: 5’ CTTCCCACAGGGTTCTCCAC 3’ | Rattus norvegicus |
| *Integrin α5* | Forward: 5’ CATGAAGGCAGGCACCAGTA 3’  Reverse: 5’ TGGGAAGGAGACCATGTTGC 3’ | Rattus norvegicus |
| *Integrin α6* | Forward: 5’ AGGTTCGAGTGACGGTGTTT 3’  Reverse: 5’ AGTGTGGATCTCAGCCTTGTG 3’ | Rattus norvegicus |
| *Integrin α7* | Forward: 5’ CCGACAGCCACTACCTCATT 3’  Reverse: 5’ TCAAGGTCAAGTCTCCGGCT 3’ | Rattus norvegicus |
| *Integrin α8* | Forward: 5’ TACAACGGAAACGCCAGAGG 3’  Reverse: 5’ CCGACAAGTAAATCTGGGTAATCA 3’ | Rattus norvegicus |
| *Integrin α9* | Forward: 5’ GCTCGAGAGAGGAATCGTGG 3’  Reverse: 5’ CATGGGGCAAGATGTGGTCT 3’ | Rattus norvegicus |
| *Integrin β1* | Forward: 5’ TGAAGTGAACAGTGAAGACA 3’  Reverse: 5’ GACCTATCGCAGTTGAAGT 3’ | Rattus norvegicus |
| *Integrin β3* | Forward: 5’ TGACCCGCTTCAATGACGAA 3’  Reverse: 5’ ATGGGTCTTGGCATCAGTGG 3’ | Rattus norvegicus |
| *Integrin β4* | Forward: 5’ TACAGATTCCCGTGGGGCTA 3’  Reverse: 5’ CGCCGTTGAGTAGCTGGTAT 3’ | Rattus norvegicus |
| *Integrin β5* | Forward: 5’ CCGAGATACCAGACCAATC 3’  Reverse: 5’ GCTTCCTCACTTCCTCATT 3’ | Rattus norvegicus |
| *Integrin β6* | Forward: 5’ TCCGCTTACGAAGAACTG 3’  Reverse: 5’ GATGCTGTATCTCCGACTT 3’ | Rattus norvegicus |
| *Integrin β8* | Forward: 5’ CGTTTGTGGCAGGAATTGTCT 3’  Reverse: 5’ TGTTTGGGCCACAGGATGTT 3’ | Rattus norvegicus |
